# Transient receptor potential Vanilloid 1-based gene therapy alleviates orthodontic pain in rats

**DOI:** 10.1038/s41368-019-0044-3

**Published:** 2019-03-11

**Authors:** Rui Guo, Yang Zhou, Hu Long, Di Shan, Jing Wen, Huimin Hu, Hong Yang, Zhouqiang Wu, Wenli Lai

**Affiliations:** 1 State Key Laboratory of Oral Diseases & National Clinical Research Center for Oral Diseases & Department of Orthodontics, West China Hospital of Stomatology, Sichuan University, Chengdu, China; 2 Jiangsu Key Laboratory of Oral Diseases, Department of Orthodontics, Stomatology Hospital Affiliated with Nanjing Medical University, Nanjing, China

**Keywords:** Genetic models, RNAi

## Abstract

Orthodontic pain that is induced by tooth movement is an important sequela of orthodontic treatment and has a significant effect on patient quality of life. Studies have shown that the high expression of transient receptor potential vanilloid 1 (TRPV1) in trigeminal ganglions plays a vital role in the transmission and modulation of orofacial pain. However, little is known about the role of TRPV1 in orthodontic pain. In this study, male Sprague–Dawley rats were randomly assigned to six groups to study the role of TRPV1 in the modulation of tooth-movement pain. The expression levels of TRPV1 mRNA and protein were determined by real-time PCR and western blot, respectively. Moreover, pain levels were assessed using the rat grimace scale (RGS). The role of TRPV1 in modulating tooth-movement pain was examined by injecting a TRPV1 antagonist into the trigeminal ganglia of rats. A lentivirus containing a TRPV1 shRNA sequence was constructed and transduced into the rats’ trigeminal ganglia. The results showed that the expression levels of TRPV1 protein and mRNA were elevated following tooth-movement pain. Pain levels increased rapidly on the 1^st^ day, peaked on the 3^rd^ day and returned to baseline on the 14^th^ day. The TRPV1 antagonist significantly reduced tooth-movement pain. The lentivirus containing a TRPV1 shRNA sequence was able to inhibit the expression of TRPV1 and relieved tooth-movement pain. In conclusion, TRPV1-based gene therapy may be a treatment strategy for the relief of orthodontic pain.

## Gene therapy: A bracing new treatment for orthodontic pain?

Gene therapy targeting a pain receptor in nerve cells carrying signals from the jaw could be an effective treatment for the pain associated with orthodontic treatment. Pain triggered by the movement of teeth is a common reason why patients terminate orthodontic treatment early. Previous studies have suggested that tooth movement is associated with an increase in the expression of transient receptor potential vanilloid 1 (TRPV1), a receptor which is activated by various physical and chemical stimuli, triggering pain. In this study, Wenli Lai at Sichuan University and colleagues confirm that TRPV1 expression increases in response to orthodontic forces which coincide with tooth movement pain in rats. They also show that a lentivirus vector can be used to deliver a small interfering RNA molecule that blocks the expression of TRPV1, alleviating pain.

## Introduction

Pain caused by tooth movement can be perceived during the entire duration of an orthodontic treatment, e.g., separator placement, initial archwire engagement, banding, wearing elastics, rapid maxillary expansion, and debonding, and it can especially be perceived during the initial phases of treatment^1^. It has been shown that 85% of orthodontic patients experience mild to moderate pain, while 9% of them endured severe pain on the first day of treatment^2,3^. This unpleasant experience is one of the main factors contributing to poor compliance and treatment termination^4,5^. Therefore, the successful management of orthodontic pain is critical in clinical practice.

To date, several approaches have been used to alleviate orthodontic pain, such as nonsteroidal anti-inflammatory drugs (NSAIDs), mechanical vibration, laser therapy, and behavioural therapy^6–12^. Unfortunately, none of these studies have shown any of these methods to be truly effective or clinically validated. Gene therapy, which is the delivery of genes to target cells to alter gene expression, is a viable and promising modality in the treatment of orthodontic pain^11^. The expression of genes to inhibit pain was upregulated in the primary afferent nerve and the dorsal horn of the spinal cord when an analgesic gene, a pain related receptor gene, or a cytokine gene were inserted into the specific transport carrier or when the recombinant vector was imported into the nervous system^13^. RNA interference (RNAi) is a powerful tool to silence gene expression or translation by neutralising targeted mRNA molecules in mammalian cells and is widely used clinically^14^. Particularly, small interfering RNA (siRNA) is more efficient at interference than traditional antisense RNA, and it has become more popular in the application of the targeted interference of gene expression and related gene functions that are involved in pain regulation^15,16^. Studies have shown in a rat neuropathic pain model that the intrathecal injection of siRNA containing the transient receptor potential vanilloid 1 (TRPV1) gene can downregulate TRPV1 expression and thereby ease pain. Successful anti-nociceptive gene therapy can be achieved by the downregulation of pro-nociceptive genes and/or upregulation of anti-nociceptive genes^17,18^.

TRPV1 has been widely recognised as a key component of both inflammatory and neuropathic pain in the sensory system^19^. It is a non-selective cation channel that is mainly distributed in the primary and secondary sensory neurons such as the trigeminal ganglia (TG)^19^. Since it is a multi-modal sensory receptor, TRPV1 can be activated by capsaicin, nociceptive thermal stimulation ( ≥ 43 °C), extracellular acidic pH, mechanical signals, and exogenous chemical stimuli^20,21^. When TRPV1 is activated by such stimuli, the resulting physiological response is pain. Pain transmitters, inflammatory mediators, and injuries can regulate the activity of TRPV1 and cause a cascade of signals to be produced in the downstream cells. It is well-documented that orthodontic tooth movement can induce an acidic extracellular micro-environment, initiate mechanical signals, and generate inflammatory mediators^6^. These nociceptive signals can activate TRPV1 and induce painful sensations^22,23^. TRPV1 expression in TG and periodontal tissues was increased following orthodontic tooth movement^24–26^. Moreover, blocking TRPV1 in periodontal tissues with its antagonist alleviated orthodontic pain^26^. All of these findings suggest that TRPV1 plays an essential role in the modulation of orthodontic pain. However, whether TRPV1-based gene therapy could alleviate tooth-movement-induced orofacial pain is poorly understood. Therefore, this study was aimed at examining whether TRPV1-based gene therapy can alleviate tooth-movement-induced orofacial pain in rats.

## Results

### The effects of orthodontic force on TRPV1 expression in the TG of rats

**Fig. 1:**
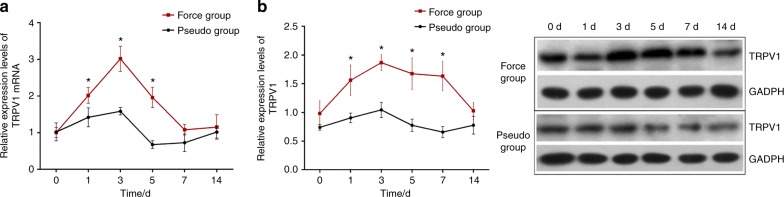
TRPV1 protein and mRNA expression in the TG of rats following orthodontic treatments, as analysed by RT-qPCR (**a**) or western blot (**b**)

As displayed in Fig. 1, TRPV1 mRNA and protein expression were increased following orofacial pain that was induced by tooth movement. A two-way ANOVA with repeated measures revealed that the TRPV1 mRNA and protein expression levels were significantly influenced by group (*P* value of 0.001 for mRNA expression and 0.004 for protein expression), time (both *P* *<* 0.001), and interactions (*P* < 0.001). Further analyses revealed that the TRPV1 protein expression levels were significantly higher in the force group than those in the pseudo-force group on the 1^st^ day (*P* < 0.001), the 3^rd^ day (*P* < 0.001), the 5^th^ day (*P* < 0.001), and the 7^th^ day (*P* < 0.001), while no differences were observed between the two groups at baseline (*P* = 0.479) or on the 14^th^ day (*P* = 0.455). Likewise, TRPV1 mRNA expression levels were significantly higher in the force group than in the pseudo group on the 1^st^ day (*P* = 0.023), the 3^rd^ day (*P* < 0.001), and the 5^th^ day (*P* < 0.001), while no differences were seen between the two groups at baseline (*P* = 0.930), on the 7^th^ day (*P* = 0.356) or on the 14^th^ day (*P* = 0.978). The details are also shown in Table 1.

**Table 1:** The effects of orthodontic force on TRPV1 protein and mRNA expression in TG of rats

Items	Time intervals/d	Force group		Pseudo-force group		*P* value
		Mean ± SD	95% CI	Mean ± SD	95% CI	
Protein expression	0	0.983 ± 0.221	0.732,1.233	0.739 ± 0.49	0.347,1.131	0.479
	1	1.560 ± 0.266	1.258,1.862	0.906 ± 0.084	0.810,1.002	< 0.001^***^
	3	1.869 ± 0.138	1.712,2.026	1.044 ± 0.132	0.895,1.193	< 0.001^***^
	5	1.675 ± 0.278	1.361,1.989	0.775 ± 0.109	0.652,0.898	< 0.001^***^
	7	1.632 ± 0.253	1.346,1.961	0.658 ± 0.098	0.546,0.770	< 0.001^***^
	14	1.027 ± 0.147	0.860,1.194	0.777 ± 0.155	0.601,0.953	0.455
mRNA expression	0	1.007 ± 0.138	0.850,1.164	1.022 ± 0.247	0.742,1.302	0.930
	1	2.017 ± 0.214	1.776,2.258	1.419 ± 0.258	1.127,1.711	0.023^*^
	3	3.016 ± 0.343	2.628,3.404	1.586 ± 0.102	1.470,1.702	< 0.001^***^
	5	1.962 ± 0.277	1.648,2.276	0.676 ± 0.104	0.558,0.794	< 0.001^***^
	7	1.081 ± 0.146	0.916,1.246	0.728 ± 0.241	0.456,1.000	0.356
	14	1.154 ± 0.334	0.776,1.532	1.018 ± 0.166	0.830,1.206	0.978

^*^P < 0.05, ^**^P < 0.01, ^***^P < 0.001

### The effects of the TRPV1 antagonist on TRPV1 expression in the TG of rats

**Fig. 2:**
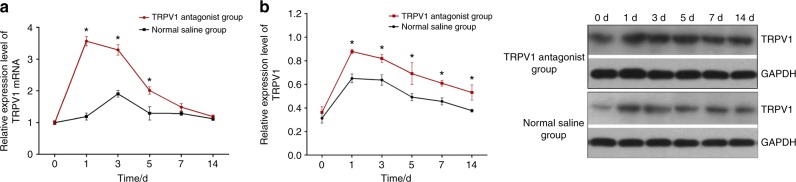
The effects of the TRPV1 antagonist SB366791 on expressions of mRNA (**a**) and protein (**b**) in rat's TG

The effects of the TRPV1 antagonist (SB366791) on TRPV1 protein and mRNA expression is shown in Fig. 2. A two-way repeated measures ANOVA revealed that TRPV1 mRNA and protein expression were significantly influenced by group (*P* value of 0.001 for mRNA expression and 0.001 for protein expression), time (both *P* < 0.001), and interaction (both *P* < 0.05). Further analyses revealed that TRPV1 protein expression levels were higher in the force + TRPV1 antagonist (SB366791) group than those in the force + normal saline group on day 1 (*P* < 0.001), day 3 (*P* < 0.001), day 5 (*P* < 0.001), day 7 (*P* = 0.002), and day 14 (*P* = 0.002). The baseline levels were similar between the two groups (*P* = 0.714), as presented in Table 2. Likewise, TRPV1 mRNA expression levels were higher in the force + TRPV1 antagonist group than in the force + saline group on day 1 (*P* <0.001), day 3 (*P* < 0.001), and day 5 (*P* < 0.001), while no statistically significant differences between the two groups were present at baseline (*P* = 0.994), on day 7 (*P* = 0.254), or on day 14 (*P* = 0.982).

**Table 2:** The effects of the TRPV1 antagonist on TRPV1 protein and mRNA expression in TG ganglia of rats

Items	Time intervals/d	Force + TRPV1 antagonist group		Force + normal saline group		*P* value
		Mean ± SD	95% CI	Mean ± SD	95% CI	
Protein expression	0	0.363 ± 0.047	0.310,0.416	0.313 ± 0.041	0.266,0.360	0.714
	1	0.830 ± 0.013	0.814,0.846	0.651 ± 0.038	0.608,0.694	< 0.001^***^
	3	0.820 ± 0.034	0.781,0.859	0.637 ± 0.044	0.586,0.688	< 0.001^***^
	5	0.691 ± 0.092	0.587,0.795	0.493 ± 0.031	0.458,0.528	< 0.001^***^
	7	0.610 ± 0.024	0.583,0.637	0.456 ± 0.030	0.423,0.489	0.002^**^
	14	0.531 ± 0.065	0.457,0.606	0.377 ± 0.012	0.363,0.391	0.002^**^
mRNA expression	0	1.001 ± 0.049	0.944,1.058	1.001 ± 0.062	0.930,1.072	0.994
	1	3.566 ± 0.150	3.395,3.737	1.190 ± 0.110	1.067,1.314	< 0.001^***^
	3	3.290 ± 0.166	3.102,3.478	1.907 ± 0.106	1.787,2.027	< 0.001^***^
	5	2.011 ± 0.115	1.880,2.142	1.291 ± 0.211	1.052,1.530	< 0.001^***^
	7	1.484 ± 0.114	1.355,1.613	1.287 ± 0.063	1.216,1.358	0.254
	14	1.190 ± 0.049	1.135,1.245	1.123 ± 0.056	1.060,1.186	0.982

^*^P < 0.05, ^**^P *<* 0.01, ^***^P *<* 0.001

### Lentivirus vector transduction

**Fig. 3:**
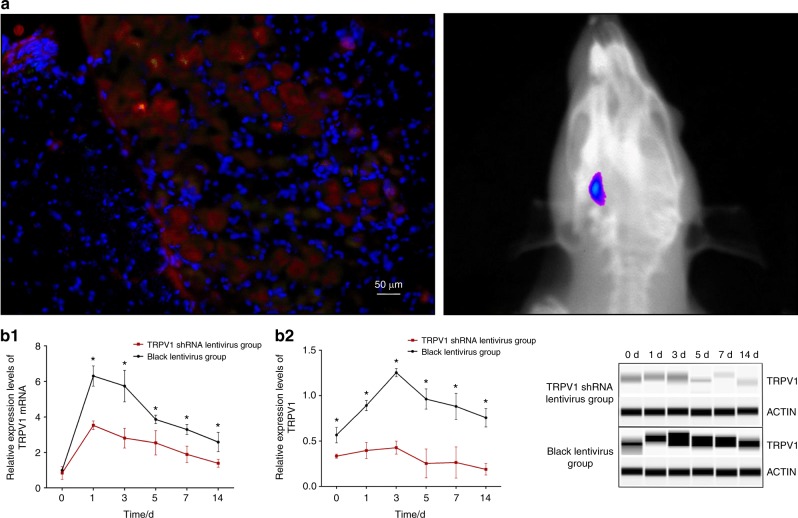
**Lentivirus vector transduction and effects of the lentiviral vector on TRPV1 expression. a** Bioluminescent signal of a TG in a rat in vivo (left), which is consistent with the TG immunofluorescence stained image radiograph (right). **b** Effects of TRPV1 shRNA lentivirus on TRPV1 mRNA (b1) and protein (b2) expression in rat's TG

As depicted in Fig. 3a, both in vivo fluorescence imaging and immunostaining showed successful lentiviral transduction of the TG. The in vivo fluorescence images also confirmed that the 100% accuracy of the TG-injection.

### The effect of the lentiviral vector on TRPV1 expression in the TG of rats

As shown in Fig. 3b, the TRPV1 mRNA and protein expression that was induced by the lentiviral vector displayed a unique pattern. TRPV1 expression decreased following orofacial pain. A two-way repeated measures ANOVA found that both the TRPV1 protein and mRNA expression levels were significantly influenced by group (*P* value of 0.001 for mRNA expression and 0.001 for protein expression), time (both *P* < 0.001), and the interactions (both *P* < 0.001). As shown in Table 3, the TRPV1 protein expression levels in the force + TRPV1 shRNA lentivirus group were significantly lower than those of the force + blank lentivirus group on the 1^st^ day (*P* < 0.001), the 3^rd^ day (*P* < 0.001), the 5^th^ day (*P* < 0.001), the 7^th^ day (*P* < 0.001), and the 14^th^ day (*P* < 0.001), except for the baseline (*P* = 0.064). The TRPV1 mRNA expression levels were also lower in the force + TRPV1 shRNA lentivirus group compared with those of the force + blank lentivirus group on the 1^st^ day (*P* < 0.001), the 3^rd^ day (*P* < 0.001), the 5th day (*P* = 0.016), the 7^th^ day (*P* = 0.009), and the 14^th^ day (*P* = 0.033), but no differences were seen between the two groups at baseline (*P* = 0.999).

**Table 3:** The effects of the lentiviral vector on TRPV1 protein and mRNA expression in the TG of rats

Items	Time intervals/d	Force + TRPV1 shRNA lentivirus group		Force + blank lentivirus group		*P* value
		Mean ± SD	95% CI	Mean ± SD	95% CI	
Protein expression	0	0.334 ± 0.023	0.309,0.359	0.567 ± 0.085	0.471,0.663	0.064
	1	0.397 ± 0.090	0.295,0.499	0.892 ± 0.056	0.829,0.955	< 0.001^***^
	3	0.428 ± 0.072	0.348,0.508	1.254 ± 0.046	1.201,1.307	< 0.001^***^
	5	0.254 ± 0.159	0.074,0.434	0.963 ± 0.111	0.838,1.088	< 0.001^***^
	7	0.265 ± 0.171	0.071,0.459	0.882 ± 0.143	0.719,1.045	< 0.001^***^
	14	0.190 ± 0.063	0.117,0.263	0.758 ± 0.100	0.644,0.812	< 0.001^***^
mRNA expression	0	0.847 ± 0.361	0.437,1.257	1.000 ± 0.018	0.978,1.022	0.999
	1	3.528 ± 0.244	3.252,3.804	6.309 ± 0.566	5.668,6.952	< 0.001^***^
	3	2.808 ± 0.544	2.181,3.435	5.742 ± 0.889	4.737,6.747	< 0.001^***^
	5	2.543 ± 0.684	1.769,3.317	3.860 ± 0.242	3.586,4.134	0.016^*^
	7	1.893 ± 0.464	1.368,2.418	3.295 ± 0.287	2.972,3.618	0.009^**^
	14	1.387 ± 0.027	1.130,1.644	2.585 ± 0.545	1.970,3.200	0.033^*^

^*^P *<* 0.05, ^**^P *<* 0.01, ^***^P *<* 0.001

### Assessment of orofacial pain

**Fig. 4:**
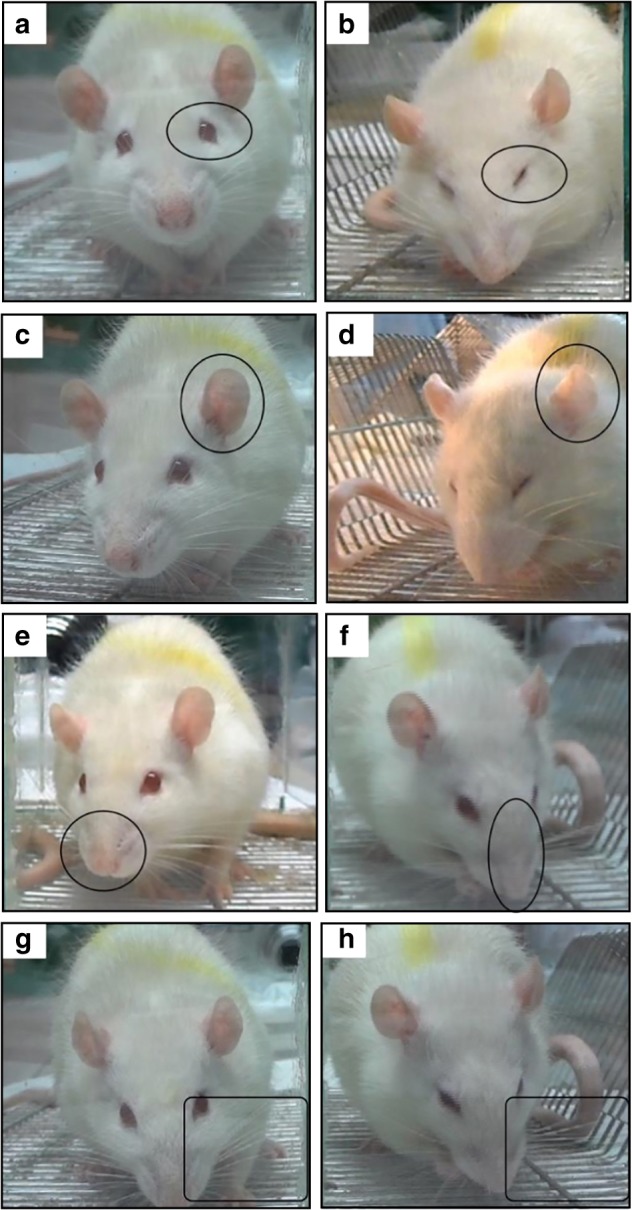
Signs of orthodontic pain expressed in the eyes (**b**), ears (**d**), mouth (**f**), and beards (**h**) in the experiment group, and their normal signs expressed in the eyes (**a**), ears (**c**), mouth (**e**), and beards (**g**) for comparison

The RGS scores, which are regarded as the surrogates for the pain levels in the rats, were coded by the images that were captured from the videotapes. Compared to the baseline of 0, the RGS scores increased in all the groups that were tested. The signs of pain in response to orthodontic treatment included a decreased width of the palpebral fissures, ears that tended to curl and angle outwards or forwards, flattened and elongated noses, and whiskers that tended to bunch and move forward (shown in Fig. 4). All of these features were observed in both the orthodontic force and pseudo-force groups but were much subtler and were seen for a shorter duration in the latter group. A two-way repeated measures ANOVA showed that the orofacial pain assessments of all groups were significantly influenced by group (*P* value of 0.001 for force group, 0.001 for antagonist group and 0.027 for lentivirus group, respectively), time (all *P* < 0.001), and the interactions (all *P* < 0.001).

**Fig. 5:**
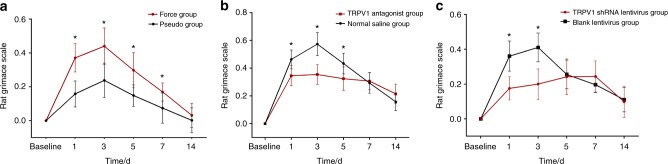
Comparison of orthodontic pain expressed as RGS between **a** force group A and pseudo-force group B, **b** force + TRPV1 antagonist group C, and force + normal saline group D, **c** force + TRPV1 shRNA lentivirus group E and force + blank lentivirus group F

The study showed that the groups with similar RGS scores shared similar TRPV1 expression trends. As displayed in Fig. 5a and Table 4, the RGS scores in the force group were significantly higher than those in the pseudo-force group on the 1^st^ day (*P* < 0.001), the 3^rd^ day (*P* < 0.001), the 5^th^ day (*P* = 0.001), and the 7^th^ day (*P* = 0.087), while similarities between the two groups were seen at baseline and on the 14^th^ day (*P* = 0.970).

**Table 4:** The effects of orthodontic force, TRPV1 antagonist and lentiviral vector on rats’ orofacial pain assessment

Items	Time intervals/d	Experiment group ^a^		Control group ^b^		*P* value
		Mean ± SD	95% CI	Mean ± SD	95% CI	
Orthodontic force	0	0.000 ± 0.000	0.000,0.000	0.000 ± 0.000	0.000,0.000	—
	1	0.370 ± 0.083	0.313,0.427	0.158 ± 0.077	0.105,0.211	< 0.001^***^
	3	0.439 ± 0.108	0.365,0.513	0.238 ± 0.101	0.167,0.309	< 0.001^***^
	5	0.297 ± 0.104	0.224,0.370	0.148 ± 0.063	0.105,0.191	0.001^**^
	7	0.169 ± 0.054	0.132,0.206	0.073 ± 0.089	0.012,0.134	0.087
	14	0.031 ± 0.071	−0.018,0.080	0.002 ± 0.074	−0.049,0.053	0.970
TRPV1 antagonist	0	0.000 ± 0.000	0.000,0.000	0.000 ± 0.000	0.000,0.000	—
	1	0.345 ± 0.071	0.288,0.402	0.463 ± 0.068	0.408,0.518	0.005**
	3	0.354 ± 0.070	0.298,0.410	0.573 ± 0.083	0.507,0.639	< 0.001***
	5	0.324 ± 0.084	0.256,0.392	0.393 ± 0.075	0.333,0.453	0.016*
	7	0.306 ± 0.063	0.256,0.356	0.293 ± 0.074	0.234,0.352	0.698
	14	0.214 ± 0.071	0.157,0.271	0.157 ± 0.063	0.106,0.208	0.111
Lentiviral vector	0	0.000 ± 0.000	0.000,0.000	0.000 ± 0.000	0.000,0.000	—
	1	0.175 ± 0.067	0.122,0.228	0.361 ± 0.086	0.292,0.430	< 0.001^***^
	3	0.200 ± 0.087	0.129,0.271	0.411 ± 0.083	0.344,0.478	< 0.001^***^
	5	0.242 ± 0.102	0.160,0.324	0.255 ± 0.083	0.188,0.322	0.999
	7	0.244 ± 0.090	0.173,0.315	0.196 ± 0.046	0.159,0.233	0.849
	14	0.098 ± 0.090	0.025,0.171	0.111 ± 0.070	0.054,0.168	0.999

^*^P < 0.05, ^**^P < 0.01, ^***^P < 0.001

^a^Experiment group in orthodontic force, TRPV1 antagonist and lentiviral vector experiment refer to force group, force + TRPV1 antagonist group, and force + TRPV1 shRNA lentivirus group, respectively

^b^Control group in orthodontic force, TRPV1 antagonist and lentiviral vector experiment refer to pseudo-force group, force + normal saline group, and force + blank lentivirus group, respectively

As for the effects of the TRPV1 antagonist SB366791 on the response of rats to the orthodontic treatments, although SB366791 upregulated TRPV1 protein and mRNA expression in the TG in this study, the RGS scores in antagonist group C were significantly higher than those in the control group D on days 1 (*P* = 0.005), 3 (*P* <0.001), and 5 (*P* = 0.016), but no differences were seen between the two groups on day 7 (*P* = 0.698) or day 14 (*P* = 0.111) (Fig. 5b).

In terms of the comparisons of the RGS score between the force + TRPV1 shRNA lentivirus group and the force + blank lentivirus group, the scores of the former group were significantly lower than those of the latter group on days 1 (*P* < 0.001) and 3 (*P* < 0.001), while no statistically significant differences were found at baseline or on day 5 (*P* = 0.999), day 7(*P* = 0.849), or day 14 (*P* = 0.999) (Fig. 5c).

## Discussion

Our study shows that TRPV1 was expressed and functional in the TG of healthy rats. The expression of TRPV1 was upregulated by orthodontic forces that coincided with tooth-movement pain in rats, indicating that TRPV1 was involved in orthodontic pain. These results were in accordance with those reported previously^24,27^. Notably, TRPV1 activation by orthodontic tooth movement directly affects TG function, which in turn modulates orthodontic pain. This hypothesis arises from: (a) the TRPV1 mRNA expression in the TG was validated by a lack of expression in rats from the negative control, the pseudo-force group, (b) the high levels of TRPV1 protein expression in the TG of rats in the force group, as shown in the western blot assays, (c) the RGS changes upon TRPV1 activation in the force group compared with either the baseline or the pseudo-force groups, and (d) this variation in pain level was consistent with the TRPV1 protein and mRNA expression in the TG, which further illustrates that changes in TRPV1 expression play an important role in the transmission and regulation of pain signals during tooth movement. Importantly, TRPV1-mediated increases in both TRPV1 mRNA and protein expression were also significantly inhibited by both the TRPV1 antagonist and the inoculation of the TRPV1 shRNA lentivirus into the TG of the rats.

Numerous novel and potent TRPV1 modulators have been identified to date, especially antagonists that can be used in pain treatment. These compounds include capsazepine, SB366791 (a cinnamide analogue), AMG-9810 (a cinnamide analogue), A-425619 (a urea analogue), BCTC (a urea analogue), and JNJ-17203212 (a urea analogue)^28–32^. All of these antagonists are small molecule compounds. Among them, SB366791 is an authorised neotype and is both a powerful and selective TRPV1 antagonist. It can competitively bind to the TRPV1 receptor and thereby effectively inhibit the TRPV1 activation process that is mediated by capsaicin, acid stimulation, pain, and burns^28^. Additionally, different viral vector types, including adenovirus, lentivirus, herpes simplex virus, and adeno-associated viruses, have been developed to transduce genes of interest into their target cells^33–36^. In particular, lentiviruses are characterised by their effective integration of RNA interference sequences against proinflammatory genes into TG and their stable gene expression, with an easier transduction of DNA into neurons and neuroglial cells^37^. Therefore, a lentivirus was used in the present study to deliver RNA interference sequences against proinflammatory genes into the TG of rats. We demonstrated that the lentivirus vector, representing a form of gene therapy, was much better than the chemical antagonist SB366791 at relieving the pain of tooth movement. Hence, owing to its high therapeutic efficacy, the application of lentivirus vectors in clinical practice will become a viable treatment strategy for orthodontic pain relief once state administrative drug authorities can regulate the quality and safety of these products and supervise how they are clinically used.

In comparison with the control group, TG inoculation with SB366791 upregulates TRPV1 mRNA and protein expression on days 1, 3, and 5 following tooth movements and alleviates the pain levels at these time intervals. These findings were inconsistent with our expectations, which could be attributed to the fact that TRPV1 synthesis occurs in a primary sensory afferent neuron, such as the dorsal root and the TG and is followed by bidirectional axonal transport to the central and peripheral axon terminals. However, the TRPV1 receptor is distributed in the primary afferent terminals that are switched off under normal conditions, and no glutamate neurotransmitters are released by TRPV1-dependent activation. The inflammatory factors that are locally generated facilitate the synthesis of TRPV1, which activates the receptor ion channels during peripheral inflammation. Conversely, protein phosphorylation is catalysed by either PKA or PKC, which are activated by the voltage-gated Ca^2+^ channel-dependent mechanism that causes the receptors to open in response to an elevation in TRPV1^38,39^. SB366791 can inhibit glutamatergic release and transmission in a subset of neurons via a presynaptic mechanism following peripheral inflammation^40^. These results provide evidence for a mechanism by which TRPV1 contributes to inflammatory pain.

The levels of TRPV1 mRNA and protein expression in the TG of the TRPV1 shRNA lentivirus group were significantly lower than those in the blank lentivirus control group at all time intervals. Moreover, the pain levels, expressed as RGS, were significantly lower in the lentivirus group on days 1 and 3, but no significant differences after day 5 were observed compared to the control group, which likely occurs because siRNA is the key RNAi molecule that affects pain relief. This gene inhibition is transient, which is quite different from gene knockout models, in which the inhibition lasts longer^41^. Lentivirus vectors that target transduction mechanisms reduced TRPV1 expression only in the TG. The body was still able to stabilise its internal environment through the nervous, immune and endocrine systems, activating other related signalling pathways or signalling molecules that can regulate neuronal excitement and affect pain transmission, ultimately changing pain perception^42^. All of the reasons mentioned above could explain the differences we observed in this experiment at the molecular or the behavioural level.

In conclusion, this study showed that TRPV1 mRNA and protein expression levels in the TG of rats, together with the RGS scores, were significantly upregulated following the orthodontic treatments, and they rapidly increased on day 1, peaked on day 3, maintained their high levels on days 5 and 7, and declined to baseline on day 14. The increases in TRPV1 mRNA and protein expression, however, were significantly inhibited by the inoculation of the TRPV1 antagonist SB366791 and the lentivirus vector that transfects RNA interference sequences against proinflammatory genes into the TG of rats, which both led to the alleviation of orthodontic pain. The TRPV1 shRNA lentivirus group showed a much higher therapeutic efficacy in the relief of orthodontic pain compared with that of the SB366791 group. Our findings suggest that the use of a TRPV1 shRNA lentivirus in clinical practice will become a viable treatment strategy for orthodontic pain relief. Future quality and safety evaluations of these vectors should be performed for clinical approval by the drug administrative authorities.

## Materials and methods

### Animals

Two hundred and sixteen male Sprague–Dawley rats, weighing between 200 g and 300 g, were obtained from the Animal Experimental Center at Sichuan University. They were maintained in the animal facility and kept in an air-conditioned room at 21 °C with a 12 h light-dark cycle. Standard rat chow and water were provided ad libitum. Animal experiments were performed in accordance with protocols that were approved by the ethical committee of the State Key Laboratory of Oral Diseases, Sichuan University (protocol No.WCCSIRB-D-2014-084).

The rats were randomly assigned to 6 groups: force group (group A; *n* = 36), pseudo-force group (group B; *n* = 36), force + TRPV1 antagonists (SB366791, positive control) group (group C; *n* = 36), force + normal saline group (group D; *n* = 36), force + TRPV1 shRNA lentivirus group (group E; *n* = 36), and force + blank lentivirus group (group F; *n* = 36). Rats were anaesthetised with an intraperitoneal injection of 7% chloral hydrate in normal saline (NS) solution at the dose of 0.06 mL·g^−1^ body weight, and then fixed Ni-Ti alloy closed-coil springs were ligated to the rats with ligation wires (0.2 mm in diameter) between the left maxillary first molar and the upper incisor of the rats to simulate orthodontic forces. In all force groups, the springs delivered a 40 g force measured, as by a force meter (Tiantian, Changsha, China), while a 0 g force was used for the pseudo-force group. Rats were euthanized by decapitation after being anaesthetised with pentobarbital sodium (50 mg·kg^−1^·bw) on days 0, 1, 3, 5, 7, and 14 (*n* = 6 for each group per day). The rats in all groups that were euthanized on day 14 were used in the orofacial pain assessments on days 0, 1, 3, 5, 7, and 14. Moreover, rats that were euthanized on day 0 did not receive any interventions and were chosen as the baseline control for each group. The details of animal use in different groups are depicted in Supplementary Fig. 1. All sections of this report adhere to the ARRIVE Guidelines for reporting animal research^43^.

### Lentivirus vector preparation

A lentivirus vector encoding an enhanced red fluorescence protein was recombined with the rat TRPV1 RNA interference sequence (sense: CAGATAACACAGTTGACAA). The recombined sequence was amplified with a polymerase chain reaction (PCR) assay. Viral vectors were packaged and harvested by transfection into 293 T cells, and this was followed by visualisation under a fluorescent microscope. The viral titre was determined using TaqMan PCR and expressed as transducing unit (TU) per mL. The viral vector titre was 1.0 × 10^9^ TU·mL^−1^. In addition, blank lentivirus vectors that did not contain the RNAi sequence were simultaneously prepared as a control.

### TG inoculation

A 15 μL aliquot of the TRPV1 antagonist solution (SB366791, Sigma-Aldrich, USA), 15 μL of a normal saline solution, 10 μL of a TRPV1 shRNA lentivirus solution (virus 3.3 × 10^5^ TU·μL^−1^, polybrene 6 × 10^−3^ TU·μL^−1^, Qiagen, USA), and 10 μL of a blank lentivirus vector (virus 3.3 × 10^5^ TU·μL^−1^, polybrene 6 × 10^−3^ TU·μL^−1^) were inoculated into the TG of animals in groups C, D, E, and F, respectively. The TRPV1 antagonist was inoculated into the TG of group C rats 30 min before spring mounting, which was followed by TG inoculations on days 1, 3, 5, 7, and 14. The group D rats were treated similarly but received normal saline TG inoculations. On each inoculation day, all of the animals in these two groups were inoculated, and six rats were sacrificed four hours after the inoculations. The TG were rapidly harvested and placed in liquid nitrogen for PCR and western blot analyses. The animals in groups E and F were inoculated with either the TRPV1 shRNA lentivirus or the blank lentivirus seven days before the orthodontic springs were applied. Six rats in each group were euthanized on days 0, 1, 3, 5, 7, and 14, and the TG were harvested.

### Immunofluorescence staining

Twelve rats that received the same inoculations as the group E rats but did not have springs inserted were euthanized, and the TG were harvested and subjected to immunofluorescence staining analyses on days 1, 3, 5, and 7 after the lentiviral inoculation. The TG specimens were embedded in a cutting compound (OCT) at an optimal temperature and cut at a 14 μm thickness along the TG macroaxis in a freezing microtome. The prepared sections were deparaffinized, rinsed with phosphate-buffered saline (PBS) three times, blocked with a 3% bovine serum albumin (BSA) solution, and incubated for 30 min after fixation in acetone for 15 min. Thereafter, the sections were stained with a primary rabbit anti-TRPV1 monoclonal antibody (1:500, Abcam) and incubated for another 45 minutes at 37 °C followed by rinsing with PBS three times for 5 min. The sections were further stained with an immunofluorescent tetramethylrhodamine (TRITC)-labelled secondary antibody (1:100), incubated for 1 h at 37 °C, rinsed with PBS and finally mounted onto slides for observation under a fluorescence microscope (DM4000B, Leica, Germany).

### Verification of TG transduction in vivo

Twelve rats were euthanized to verify the transduction of the viral vectors into the TG using an in vivo fluorescence imaging system (In-Vivo Xtreme, Bruker, Germany) on days 1, 3, 5, and 7 after TRPV1 shRNA lentivirus inoculation and prior to the application of the orthodontic force springs. All rats were routinely imaged on days 1, 3, 5, and 7 after lentivirus inoculation, and three rats were imaged on each day. Cherry was selected as the fluorescence reporter gene, and it has a fluorescence detector with excitation/emission wavelengths of 587/610 nm.

### Orofacial pain assessment

For the animals in groups C, D, E, and F that were sacrificed on day 14, orofacial pain levels were assessed using the RGS between 7:00 pm and 9:00 pm on days 0, 1, 3, 5, and 7 in a room with a level of background noise less than 45 dB following the protocols described previously^44^. Briefly, the tested rats were placed individually into transparent cubicles with a 20.0 cm × 10.5 cm × 9.0 cm volume. After acclimation to the environment for 15 min, the rats were continuously videotaped for 30 min. For each rat on each assessment day, ten facial expression images were extracted every 3 min to be used for the RGS scoring. The RGS was scored independently by two analysts by examining the facial expression changes in the orbits, nose, ears, and whiskers of the rats. The two analysts’ scores of the pain levels were averaged. For each rat, the RGS scores that were determined before the interventions were applied were used as the baseline RGS. Differences in the RGS scores between the baseline and experimental tooth-movement groups were regarded as the surrogate pain levels for each rat at each assessment time point.

### The real-time RT-PCR assay

Total RNA was extracted from the TG using the Takara MiniBEST Universal RNA Extraction Kit (Takara, Shiga, Japan) according to the manufacturer’s protocols. Then, cDNA was reverse transcribed using the M-MLV test kit (M1705, Promega) following the manufacturer’s recommendations.

Rat TRPV1 (NM_031983) mRNA expression was quantified in the TG samples using triplex RT-PCR performed in a LightCycler480 (Roche, Switzerland) RT-PCR platform with the SYBR Premix Ex Taq (Perfect Real-time, TAKARA, Dalian, China) according to the manufacturer’s protocol. To quantify the amount of specific mRNA expression in the samples, a standard curve was generated for normal rat TG samples. GAPDH served as an internal standard. PCR was performed using specific primers for rat GAPDH (forward primer: TTCAACGGCACAGTCAAGG, reverse primer: CTCAGCACCAGCATCACC, expected size: 114 bp) and TRPV1 (forward primer: AAGGATGGAACAACGGGCTAG, reverse primer: TCCTGGTAGTGAAGATGTGGG, expected size: 127 bp). The final reaction volume of 12 µL consisted of 6 µL of the SYBR Premix Ex Taq, 0.3 μL of the 5 µmol·L^−1^ primer mix, 0.6 μL of the reverse transcription nucleic acid template, and 5.1 μL of RNase-free H_2_O. The thermal profile was set at 95 °C for 2 min and 94 °C for 10 s, followed by 40 cycles at 60 °C for 30 s and 40 cycles at 60 °C for 10 s. Relative mRNA transcript level calculations were performed using the comparative CT method (ΔΔCT). The average CT values of the three complex holes for each sample were calculated and marked as CT_TRPV1_ and CT_GAPDH_, ΔΔCT = N(CT_TRPV1_ − CT_GAPDH_) − (CT_TRPV1_ − CT_GAPDH_).

### Western blot analysis

The TG tissues were cut into pieces and homogenised with the RIPA lysis buffer plus phenylmethanesulfonyl fluoride (PMSF) at a ratio of 20 mg to 150–250 μL, resulting in a final concentration of 1 mm. The homogenate was incubated on ice for 30 min followed by centrifugation at 14 000 × *g* for 5 min. The supernatant was stored at −80 °C before analysis. The lysates were run on SDS-PAGE and separated by electrophoresis (Tanon, Shanghai, China). Proteins were transferred onto polyvinylidene fluoride (PVDF) membranes and blocked with 5% skim milk in a Tris-buffered saline with Tween 20 (TBST) solution for 1 h at room temperature. The sealed PVDF membranes were incubated in the primary TRPV1 goat polyclonal antibody (1:200, Santa Cruz, USA) for 2 h at room temperature, washed with TBST four times and incubated with the second HRP-conjugated goat anti-rabbit antibody (Beyotime Biotechnology, China) for 1 h. The protein blot densities were analysed using ImageJ Software (National Institutes of Health, Bethesda), which marked the target intensity band as A_TRPV1_ and the internal reference intensity band (GAPDH) as A_GAPDH_; protein expression at the corresponding time point = A_TRPV1_/A_GAPDH_.

### Statistical analyses

Statistical analyses were performed using SPSS 16.0 (SPSS, Chicago, Illinois, USA) and GraphPad Prism 6.0 software (GraphPad Software, San Diego, USA). The results are depicted as the means ± the standard deviation (SD). A one-way ANOVA (Bonferroni post hoc test) was employed to analyse the differences between TRPV1 expression and orofacial pain among different time intervals in each group. A two-way ANOVA with repeated measures was used to examine the effects of time (0, 1, 3, 5, 7, and 14 d), groups (the force group vs. the pseudo-force group, the force + TRPV1 antagonist group vs. the force + normal saline group, the force + TRPV1 shRNA lentivirus group vs. the force + blank lentivirus group), and the interactions with both TRPV1 expression and orofacial pain. For the two-group comparisons at each time interval, the Bonferroni post hoc test was used if the pretest for normality was not rejected at the 0.05 significance level. *P* values less than 0.05 were considered statistically significant.

## Supplementary information

supplementary figure 1
