# Supplementary figures and images for: Transient receptor potential Vanilloid 1-based gene therapy alleviates orthodontic pain in rats
Source: Int J Oral Sci. 2019 Mar 11;11(1):11. doi: 10.1038/s41368-019-0044-3 (PMC6409362; doi:10.1038/s41368-019-0044-3)

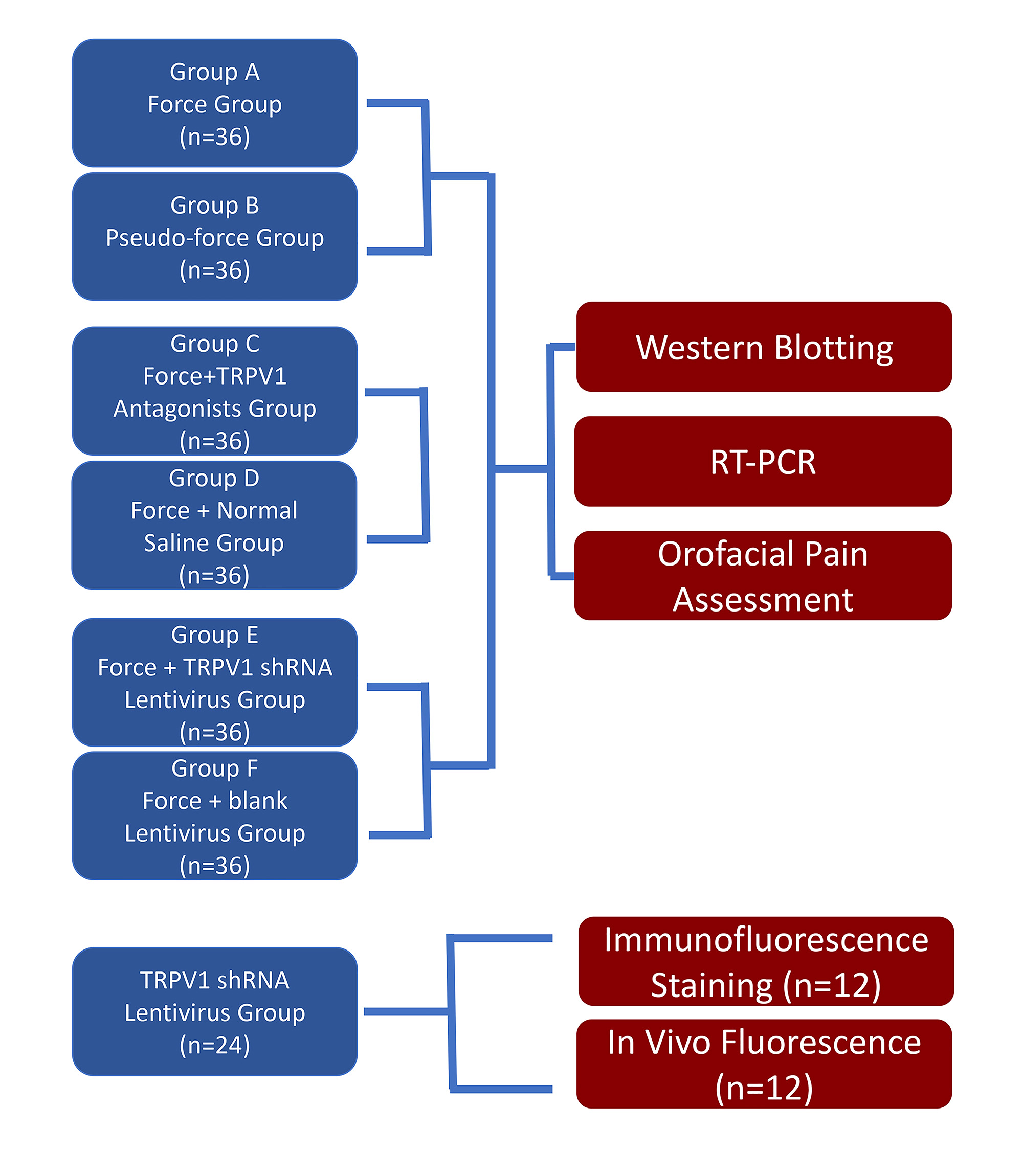

Supplement: Supplementary file 1 — supplementary figure 1 [file 41368_2019_44_MOESM1_ESM.jpg]
